# 固相萃取-超高效液相色谱-串联质谱法检测保健食品中9种人参皂苷

**DOI:** 10.3724/SP.J.1123.2020.04028

**Published:** 2021-05-08

**Authors:** Shudong CHEN, Rui FENG, Xiaojia LIN, Tujin LIANG, Qiuting HE

**Affiliations:** 1.广东省中药研究所, 广东 广州 510640; 1. Guangdong Institute of Traditional Chinese Medicine, Guangzhou 510640, China; 2.广东医科大学, 广东 东莞 523808; 2. Guangdong Medical University, Dongguan 523808, China; 3.广州检测认证集团有限公司, 广东 广州 511447; 3. Guangzhou Inspection Testing and Certification Group Co., Ltd., Guangzhou 511447, China

**Keywords:** 超高效液相色谱-串联质谱, 固相萃取, 人参皂苷, 保健食品, ultra performance liquid chromatography⁃tandem mass spectrometry (UPLC MS/MS), solid phase extraction (SPE), ginsenosides, health foods

## Abstract

建立了以固相萃取结合超高效液相色谱-串联质谱(UPLC-MS/MS)同时检测保健食品中9种原人参二醇型和原人参三醇型人参皂苷的方法。保健食品中人参皂苷经过提取后,通过Alumina-N/XAD-2 SPE柱净化,在Hypersil Gold C18色谱柱(100 mm×2.1 mm, 1.9 μm)上分离,利用乙酸铵溶液(含0.1%甲酸)和乙腈作为流动相进行梯度洗脱,采用负离子扫描,多反应监测模式测定,外标法定量。研究通过对不同填料的固相萃取小柱的考察,最终选择了Alumina-N/XAD-2复合填料,其能对保健食品复杂基质中的人参皂苷进行有效富集和净化;通过考察人参皂苷的电离裂解过程,确定人参皂苷一级质谱准分子离子和相应的碎片离子,并经过色谱条件的优化,使质谱条件下一级质谱准分子离子和相应的碎片离子均一致的3种原人参二醇型人参皂苷Rb2、Rb3、Rc同分异构体实现完全分离。结果表明,9种人参皂苷在0.005~0.5 μg/mL范围内具有很好的线性关系,相关系数均大于0.9950。方法的加标回收率为81.1%~114.2%,相对标准偏差为0.4%~8.0%。所建立的方法采用XAD-2大孔吸附树脂和中性氧化铝的复合固相萃取材料,保健食品经过简单提取可直接作为固相萃取的上样溶液进行人参皂苷的富集和净化,通过超高效液相色谱-串联质谱不仅缩短了分析时间,也能对复杂基质样品中含量相对较低的人参皂苷进行准确定性和定量。该方法通量高,简单快速,重复性好,适用于保健食品中9种人参皂苷的定性和定量分析。

## Abstract (en)

Ginsenosides are the main active compounds of ginseng, American ginseng and Panax notoginseng. They have certain pharmacological effects on the cardiovascular, immune and central nervous systems. Most ginsenosides are naturally classified as protopanaxatriol (PPT), protopanaxadiol (PPD), and oleanolic acid (OA) according to their aglycone skeletons. The nine main ginsenosides are Rb1, Rb2, Rb3, Rc, Rd, Re, Rf, Rg1 and Rg2. Accurate quantification of ginsenosides is imperative because they are the characteristic components and quality evaluation indicators of health foods. A new method based on solid phase extraction-ultra performance liquid chromatography-tandem mass spectrometry (SPE-UPLC-MS/MS) was developed for the determination of the nine ginsenosides in health foods. First, the pretreatment conditions were optimized. With the aim of purifying the samples and removing impurities, SPE cartridges with different packing materials, such as Alumina-N/XAD-2 SPE Cartridge, C18 and HLB were investigated. Based on the purification efficiencies, recoveries and other factors, the Alumina-N/XAD-2 SPE cartridge composite SPE column was selected as the pretreatment purification column. The eluents were then optimized. When water was used as the eluent, the ginsenosides could remain adsorbed on the SPE column, and could not be eluted down with other water-soluble substances. By increasing the proportion of ethanol in the eluent, the ginsenoside adsorbed on the filler of the SPE column could be gradually eluted. When the proportion of ethanol in the eluent reached 70%, the ginsenosides could be completely eluted. The effects of different volumes of 70% ethanol elution solvent (5-30 mL) on the extraction efficiencies of ginsenosides were also investigated. The results showed that when the volume of the elution solvent reached 20 mL, the ginsenosides were completely eluted. Then, the chromatographic conditions and MS parameters were optimized. By examining the ionization cracking of ginsenosides, the quasi-molecular ions and corresponding fragment ions in ginsenoside primary MS were determined. After optimizing the chromatographic conditions and MS parameters, not only the sensitivity of the method was improved, but also the isomers Rb2, Rb3 and Rc with the same quasi-molecular ions and the corresponding fragment ions were completely separated. Good separation was achieved for the nine ginsenosides, thus meeting the requirements for accurate quantification. Finally, chromatographic separation was achieved on a Hypersil Gold C18 column (100 mm×2.1 mm, 1.9 μm) under linear gradient elution using a 5 mmol/L ammonium acetate solution (with 0.1% formic acid) and acetonitrile as the mobile phases. The nine ginsenosides were detected using a triple quadrupole MS detector under ESI ^-^ and multiple reaction monitoring (MRM) modes, and quantified by the external standard method. The nine ginsenosides showed a strong positive linear correlation (*r* ^2^>0.9950) in the range of 0.005-0.5 μg/mL. The sample recoveries and the corresponding relative standard deviations (RSDs) were 81.1%-114.2% and 0.4%-8.0% (*n*=6), respectively. Eleven batches of health foods on the market, among which six batches contained ginseng, American ginseng or Panax notoginseng ingredients, were analyzed by the developed method, and the ginsenosides were detected. The total ginsenosides contents were close to those mentioned on the label. However, the nine ginsenosides were detected in one batch of health food, whose label did not indicated ginseng, American ginseng or Panax notoginseng. The nine ginsenosides were not detected in the remaining batches of health foods.The health food extract was directly loaded and purified without any complex pretreatment. The UPLC⁃MS/MS method, not only helped shorten the analysis time, but also accurate quantification of low ginsenoside contents in complex matrix samples. The developed method is simple and rapid, with high throughput, thus being suitable for the quantitative analysis of the nine ginsenosides in health foods.

人参、西洋参和三七等五加科(Araliaceae)植物不仅是传统的名贵中药材,作为纳入可用于保健食品管理的中药品种,在保健食品中的应用也非常广泛。据统计,在2006~2015年间,中国注册的保健食品中,有近40%的保健食品含有人参、西洋参或三七成分^[1]^。人参皂苷是人参、西洋参和三七的主要活性化合物,对心血管系统^[2]^、免疫系统和中枢神经系统^[3,4]^都具有药理作用,作为保健食品的特征成分和质量评价指标,准确检测其含量具有非常重要的意义。人参皂苷属于四环三萜类化合物,现已发现的种类有50多种,主要分为原人参二醇型皂苷(PPD)、原人参三醇型皂苷(PPT)和齐墩果酸型皂苷3种类型^[5]^。其中,人参皂苷Rb1、Rb2、Rb3、Rc、Rd、Re、Rf、Rg1和Rg2等9种化合物的含量超过90%^[6,7]^,本文以9种主要人参皂苷成分为研究对象,旨在建立一个快速、可靠的人参皂苷检测方法,用于保健食品中人参皂苷成分的检测和质量评价。

## 1 实验部分

### 1.1 仪器、试剂与材料

TSQ Quantum Access MAX三重四极杆质谱仪、UltiMate 3000超高效液相色谱仪(美国Thermo Scientific公司); Sartorius BSA224S-CW电子天平(德国Sartorius公司); 5424R冷冻离心机(德国Eppendorf公司); MIX-1振荡涡旋混合器(上海托莫斯科学仪器有限公司); KQ-250E超声波水浴器(昆山市超声仪器有限公司); Milli-Q Advantage A10超纯水系统(美国Millipore公司)。

乙酸铵(色谱纯,上海Macklin公司);甲醇、乙腈、甲酸(色谱纯,德国Merck公司); Alumina-N/XAD-2 SPE柱(1 g/4 g, 10 mL)和人参皂苷Rb2(批号Z99800205)、Rb3(批号Y8650010)、Rc(批号U9330025)、Rd(批号R7880050)、Rf(批号T7810020)、Rg2(批号47560010)购于上海安谱实验科技股份有限公司;对照品人参皂甙Re(批号110754-201827)、Rg1(批号110703-201731)、Rb1(批号110704-201827)购于中国食品药品检定研究院。11批保健食品均为市售。

### 1.2 标准溶液的配制

分别准确称取各目标物标准品0.01 g,用甲醇溶解并配制成1 mg/mL的标准储备液,于-18 ℃保存。分别吸取一定量的标准储备液,用甲醇稀释,涡旋混匀,配制成质量浓度为1.0 μg/mL的混合标准溶液,于-18 ℃保存。移取适量混合标准溶液,用甲醇-水(30∶70, v/v)配制成0.005~0.5 μg/mL的系列混合标准工作液,现用现配。

### 1.3 样品前处理

1.3.1 提取

固体试样:取片剂或胶囊内容物研成粉末,称取0.5 g试样,置于50 mL塑料离心管中,加入约15 mL纯化水涡旋振摇混匀,超声提取(功率300 W,频率40 kHz)30 min,以5000 r/min离心5 min,上清液转移至50 mL量瓶中,残渣用30 mL纯化水分2次重新提取,提取液并入量瓶中,用纯化水定容至刻度,混匀,待净化。

液体试样:称取2 g试样,置于50 mL塑料离心管中,加入约40 mL纯化水,涡旋振摇混匀,超声提取(功率300 W,频率40 kHz)30 min,用纯化水定容至50 mL,混匀,待净化。

1.3.2 净化

分别用20 mL 70%乙醇水溶液和20 mL水对固相萃取柱进行活化,取2.0 mL样品提取溶液上样,分别用20 mL水进行淋洗,用20 mL 70%乙醇水溶液进行洗脱,收集洗脱液;洗脱液用甲醇-水(30∶70, v/v)定容至25.0 mL,混匀后用0.22 μm混合型滤膜过滤,上机检测。若样品中人参皂苷浓度超出线性范围,用甲醇-水(30∶70, v/v)适当稀释。

### 1.4 分析条件

1.4.1 色谱条件

色谱柱:Hypersil Gold C18色谱柱(100 mm×2.1 mm, 1.9 μm)柱;柱温:40 ℃;流动相:A为5 mmol/L乙酸铵溶液(含0.1%甲酸), B为乙腈;流速:0.4 mL/min。梯度洗脱条件:0~4.0 min, 81%A; 4.0~6.0 min, 81%A~79%A; 6.0~8.0 min, 79%A~72%A; 8.0~15.0 min, 72%A~69%A; 15.0~20.0 min, 69%A~54%A; 20.0~20.5 min, 54%A~10%A; 20.5~22.0 min, 10%A; 22.0~22.5 min, 10%A~81A%。进样量:5 μL。

1.4.2 质谱条件

离子源:电喷雾电离源,负离子模式(ESI^-^);扫描方式:多反应监测(MRM)模式;离子传输毛细管温度:350 ℃;电喷雾电压:3000 V;蒸发温度:400 ℃;碰撞气体压力:0.2 Pa;辅助气流量:3.6 mL/min;鞘气流量:10.5 mL/min。9种分析物的监测离子对(Q1/Q3 ion pairs)、碰撞能量和管透镜补偿电压(tube lens)见表1。

**表 1:** 9种分析物的质谱参数

No.	Com- pound	*t*_R_/ min	Q1/Q3 ion pairs (*m/z*)	Collision energy/ eV	Tube lens/ V
1	Rg1	8.01	799.5/637.5^*^, 799.5/475.5	26, 33	150
2	Re	8.19	945.5/637.4^*^, 945.5/475.5	51, 36	180
3	Rf	11.82	799.5/475.4^*^, 799.5/637.4	36, 30	150
4	Rg2	13.86	783.5/475.4^*^, 783.5/637.4	36, 26	150
5	Rb1	15.13	1153.6/1107.7^*^, 1153.6/221.1	28, 56	180
6	Rc	16.32	1077.6/783.5^*^, 1077.6/945.5	41, 40	180
7	Rb2	17.42	1077.6/783.5^*^, 1077.6/945.5	46, 38	175
8	Rb3	17.69	1077.6/783.5^*^, 1077.6/945.5	42, 41	180
9	Rd	18.63	945.5/783.5^*^, 945.5/621.4	36, 40	164

* Quantitative ion pair.

## 2 结果与讨论

### 2.1 质谱条件优化

实验在ESI^-^和MRM模式下检测人参皂苷,由于9种人参皂苷的主要分子结构类似(见图1),人参皂苷的电离裂解过程为皂苷分子M丢失一个质子形成[M-H]^-^准分子离子,准分子离子在碰撞池与碰撞气分子发生碰撞而裂解成的碎片离子也多以丢失各种糖基G后形成[M-H-G]^-^为主^[19,20]^,离子形成和裂解机理基本一致,碎片离子具有各型皂苷的典型特征。原人参二醇型皂苷作为准分子离子,断裂后以*m/z* 783.5和*m/z* 945.5的碎片离子为主;原人参三醇型皂苷准分子离子,断裂后出现*m/z* 475.5和*m/z* 637.4为主的碎片离子。在确定了一级质谱准分子离子峰和相应的碎片离子后,实验通过对喷雾电压、碰撞能量和管透镜补偿电压等条件进一步优化,使得方法的灵敏度进一步得到提升。

**图 1:**
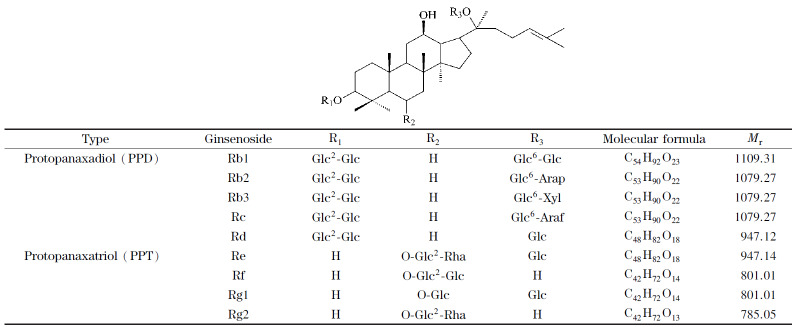
9种人参皂苷的化学结构

### 2.2 色谱条件优化

由于9种人参皂苷的分子结构相类似,通过对比可以发现,原人参二醇型结构的人参皂苷Rb2、Rb3和Rc互为同分异构体,分子结构相同,只是在空间结构上存在差别,3种物质的一级准分子离子峰和裂解失去糖基后形成的离子碎片均相同(*m/z* 1077.6/783.5、*m/z* 1077.6/945.5),且在色谱柱上的保留时间接近,因此在色谱分离时,这3种物质需达到完全分离才能准确定量。研究采用Hypersil Gold C18色谱柱(100 mm×2.1 mm, 1.9 μm),在0.4 mL/min的流速条件下,通过优化梯度洗脱条件,使人参皂苷Rb2、Rb3和Rc 3种目标物质实现完全分离,全部9种目标物质均实现较好的分离效果(见图2)。实验进一步考察了相同梯度洗脱条件下甲醇-水和乙腈-水体系流动相对9种目标物质的分离效果,结果表明,乙腈-水体系流动相的洗脱能力优于甲醇-水体系,且峰形对称性更好。在质谱条件优化过程中发现,在乙腈-水流动相体系中加入甲酸能够提高皂苷物质的响应信号,另外,添加一定比例的乙酸铵,也能改善流动相的洗脱能力和部分目标物质的峰形。经过对乙酸铵和甲酸加入量的优化,最终确定采用5 mmol/L乙酸铵溶液(含0.1%甲酸)-乙腈流动相作为实验最终条件,能获得更好的洗脱能力和信号强度。

**图 2:**
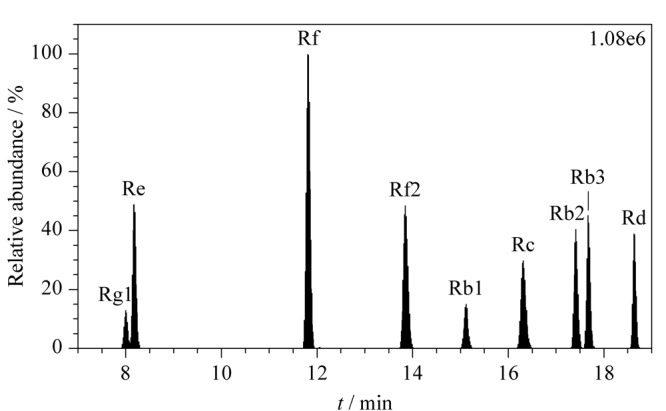
9种人参皂苷的总离子流色谱图

### 2.3 前处理条件优化

2.3.1 固相萃取柱的选择

保健食品成分相对复杂,实验的关键步骤是对目标物质的净化和富集。在传统的SPE提取净化方法中,C18固相萃取柱^[21]^和HLB固相萃取柱^[22]^是常用的固相萃取柱类型。为考察不同固相萃取方式对复杂样品净化效果的影响,实验以胶囊样品为研究对象,以基质效应(ME)的大小和样品的回收率为指标,对C18、HLB、Alumina-N/XAD-2 3种不同固相萃取柱的净化效果进行比较(见表2)。其中,基质效应以基质标准曲线各浓度点的响应值与系列混合标准工作液中相应浓度点的响应值的比值计算,ME值越接近100%,表明基质效应越低。结果表明,经Alumina-N/XAD-2 SPE Cartridge复合固相萃取柱净化后,9种目标物的基质效应明显低于C18和HLB柱净化后,回收率与另外两种萃取柱相比也有一定的优势。

**表 2:** 采用不同固相萃取柱时9种人参皂苷的基质效应和回收率

Compound	MEs/%				Recoveries/%			
	C18	HLB	Alumina-N/XAD-2		C18		HLB	Alumina-N/XAD-2
Rg1	55.2	68.1	91.5		65.6		88.2	101.4
Re	48.6	72.2	93.3		68.9		79.3	107.6
Rf	51.5	70.3	89.6		75.5		95.6	96.7
Rg2	62.3	89.1	94.4		69.4		117.9	93.5
Rb1	46.5	82.6	95.3		72.5		124.8	89.7
Rc	54.2	74.1	89.9		70.4		94.2	90.6
Rb2	55.3	63.3	90.1		78.6		90.4	98.4
Rb3	63.9	79.4	91.7		59.4		80.1	103.4
Rd	57.6	66.5	92.3		69.1		78.5	94.5

2.3.2 提取溶剂、淋洗和洗脱剂的选择

研究采用Alumina-N/XAD-2复合固相萃取柱作为前处理的净化柱,柱填料分别为XAD-2大孔吸附树脂和中性氧化铝,XAD-2大孔树脂是一种适用范围广泛的固相萃取填料,通过分子排阻作用对保健食品中的糖类、蛋白质和添加剂进行洗脱^[23]^,中性氧化铝填料能够对皂苷类、黄酮类水溶性成分或极性化合物有较强的吸附能力^[24]^,两种不同填料的复合使用能够对目标物质进行富集并与其他干扰物质分离。水饱和的正丁醇溶液或甲醇水溶液是常见的人参皂苷提取剂,在实际操作中发现,正丁醇或甲醇会洗脱固相萃取小柱XAD-2填料表面吸附的人参皂苷成分,需要在固相萃取上柱前先蒸干除去提取液中的醇类,再进行固相萃取富集和提取。实验以水饱和正丁醇溶液、甲醇-水(50∶50, v/v)溶液和纯水为提取溶液,分别对含有9种人参皂苷的保健食品进行提取,比较3种提取溶液的提取效率。结果表明,3种不同提取溶液对9种人参皂苷的提取效率基本一致,说明纯水可以对人参皂苷进行有效提取。因此,选择纯水作为提取溶剂。

另外,洗脱剂的选择和用量是前处理的关键,选择最佳洗脱剂需要兼顾人参皂苷的洗脱率、杂质洗脱率以及洗脱剂的用量。大孔树脂洗脱时常用不同体积分数的乙醇溶液,乙醇的体积分数和洗脱体积对待分离成分的含量具有较大的影响^[25]^。通过提高洗脱液中乙醇的比例能够使填料中吸附的人参皂苷逐渐被洗脱,实验发现当洗脱剂中乙醇比例达到70%时,人参皂苷基本能够完全洗脱,故选择采用70%乙醇作为洗脱剂。方法考察了不同体积(5~30 mL)洗脱剂对人参皂苷提取率的影响(见图3)。结果表明,洗脱剂达到20 mL时,基本能使人参皂苷完全洗脱。

**图 3:**
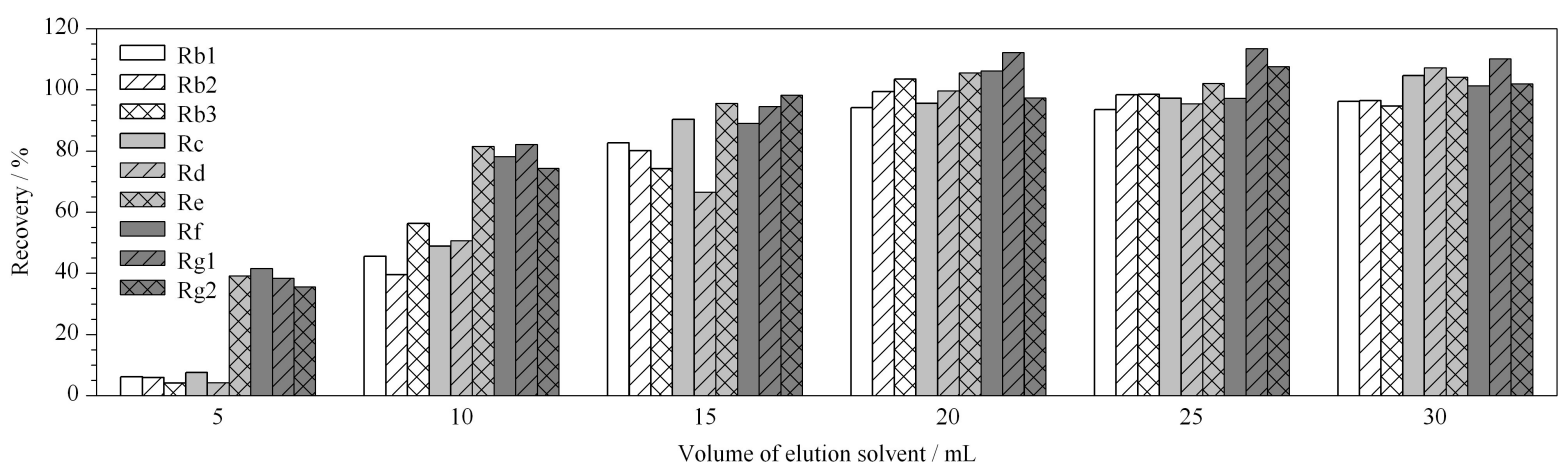
不同体积的洗脱剂对9种人参皂苷提取率的影响

### 2.4 方法学考察

2.4.1 线性范围与检出限

在优化后的色谱条件下,考察了9种人参皂苷在0.005~0.5 μg/mL范围内的线性关系,以各分析物的质量浓度为横坐标,以其相应的色谱峰面积为纵坐标,得到各自的线性方程。结果显示,9种人参皂苷在线性范围内呈现出良好的线性关系,相关系数(*r*^2^)为0.9963~0.9994。9种目标物在液体样品和固体样品中的定量限分别为2.5 mg/kg和10 mg/kg。该法灵敏度较高,可较好的应用于保健食品中9种人参皂苷的检测。

2.4.2 回收率与精密度

按照确定的前处理方法,对保健食品基质进行3个水平的加标回收试验,添加水平为定量限的1、6和12倍,每个添加水平重复6次,计算回收率和精密度。结果表明,9种人参皂苷的回收率为81.1%~114.2%,精密度为0.4%~8.0%(见表3)。说明方法可满足实验室日常检测的要求。

**表 3:** 9种人参皂苷的线性方程、相关系数、加标回收率和精密度(*n*=6)

Compound	Linear equation	*r* ^2^	10 mg/kg			60 mg/kg			120 mg/kg		
			Recovery/%	RSD/%		Recovery/%	RSD/%		Recovery/%		RSD/%
Rg1	*Y*=6.68×10^6^*X*+4.49×10^5^	0.9994	97.1	1.0		102.5	3.5		111.8		1.5
Re	*Y*=1.66×10^7^*X*+2.17×10^6^	0.9970	108.6	8.0		97.2	1.2		110.3		0.8
Rf	*Y*=5.14×10^6^*X*+6.44×10^6^	0.9963	114.2	1.8		97.5	2.3		106.2		0.8
Rg2	*Y*=4.21×10^6^*X*+3.09×10^6^	0.9991	109.8	4.7		91.6	0.9		97.2		1.8
Rb1	*Y*=1.39×10^6^*X*+1.82×10^6^	0.9969	81.4	5.7		85.1	3.3		89.1		4.4
Rc	*Y*=2.16×10^6^*X*+1.61×10^6^	0.9989	104.5	3.6		92.5	0.4		96.6		1.8
Rb2	*Y*=1.98×10^6^*X*+1.90×10^6^	0.9983	106.7	3.0		93.7	3.6		100.7		0.6
Rb3	*Y*=1.94×10^6^*X*+1.93×10^6^	0.9984	102.7	4.8		95.7	3.0		101.8		0.5
Rd	*Y*=1.87×10^6^*X*+2.51×10^6^	0.9965	108.3	1.9		92.3	3.7		103.5		2.3

*Y*: peak area; *X*: mass concentration, μg/mL.

2.4.3 稳定性

实验考察了9种人参皂苷经提取后,在样品瓶中放置1、4、8、12、24、48 h后的稳定性。结果表明,9种人参皂苷含量的RSD值≤8.66%,说明该条件下样品的稳定性良好,能满足测定要求。

2.4.4 基质效应

分别选取3种代表性基质(片剂、胶囊和口服液)的保健食品,采用1.3节的前处理方法,比较了3种代表性样品基质对9种人参皂苷测定的影响。结果显示,9种人参皂苷的基质效应在片剂样品、胶囊样品和口服液样品中分别为92.2%~102.3%、89.6%~95.3%和93.3%~105.1%。说明样品基质经过前处理过程的净化和较大体积的稀释,基质效应对化合物的影响较小,采用甲醇-水(30∶70, v/v)溶剂配制的标准溶液能够满足测定要求。

### 2.5 实际样品检测

应用所建立的方法分别对市售11批保健食品进行分析,其中6批原材料标识含有人参、西洋参、三七等五加科植物成分(样品1-6), 5批未标识含有人参、西洋参、三七等五加科植物成分(样品7-11),分别按照优化后的前处理和分析条件处理,结果见表4。

**表 4:** 保健食品样品中9种人参皂苷成分的测定结果

Sample	Rg1	Re	Rf	Rg2	Rb1	Rc	Rb2	Rb3	Rd	Total
1	0.0874	0.202	ND	0.0358	0.0162	0.0529	0.129	0.275	0.197	1.00
2	0.0700	0.214	<0.0010	0.00144	0.175	0.0460	0.00977	0.0112	0.104	0.63
3	0.229	0.0184	<0.0010	0.00341	0.0671	<0.0010	ND	<0.0010	0.0424	0.56
4	0.417	0.0549	<0.0010	0.0178	0.149	<0.0010	<0.0010	<0.0010	0.0985	0.74
5	0.0746	0.245	ND	0.0336	0.233	0.0914	0.0206	0.0280	0.126	0.85
6	0.201	0.510	ND	0.0640	0.0469	0.0950	0.284	0.770	0.709	2.68
7	ND	ND	ND	ND	ND	ND	ND	ND	ND	ND
8	0.140	0.153	0.00114	0.00915	0.00910	0.0370	0.0376	0.0242	0.0438	0.46
9	ND	ND	ND	ND	ND	ND	ND	ND	ND	ND
10	ND	ND	ND	ND	ND	ND	ND	ND	ND	ND
11	ND	ND	ND	ND	ND	ND	ND	ND	ND	ND

ND: not detected.

结果表明,标识含有五加科植物配料的6批保健食品均检出人参皂苷成分,9种人参皂苷含量占0.56%~2.68%,与样品标签标注总皂苷含量接近;有1批配料表仅标识葛根提取物、苦瓜提取物等成分的保健食品检出包括人参皂苷Rf在内的9种人参皂苷成分(样品8), 9种人参皂苷含量占0.46%;另外4批未标识含有五加科植物成分样品均未检出9种人参皂苷。

## 3 结论

本文建立了一种快速、高效分析保健食品中9种人参皂苷的超高效液相色谱-串联质谱分析方法。该法能快速测定人参提取物中9种人参皂苷的含量,通过对色谱和质谱参数、样品提取和净化方式、淋洗和洗脱剂等条件进行了优化。结果表明,该方法快速准确,灵敏度高,重复性好,实用性强,为保健食品中人参皂苷的检测及质量控制提供了可靠的技术支持。
